# ACC representations of reward-driven motivation over hierarchically-organized behavior

**DOI:** 10.1016/j.neuroimage.2025.121380

**Published:** 2025-08-15

**Authors:** Emmanouela Foinikianaki, Iris Ikink, Thomas R. Colin, Ricardo J. Alejandro, Clay B. Holroyd

**Affiliations:** Department of Experimental Psychology, Ghent University, Ghent, Belgium

**Keywords:** Anterior cingulate cortex (ACC), Hierarchical control, Cognitive control, Representational similarity analysis (RSA), Multi-voxel Pattern Analysis (MVPA), Recurrent neural network (RNN)

## Abstract

The neural mechanisms of reward-guided behavior have been researched extensively but how rewards affect the neural processes underlying the production of hierarchically-organized sequential behaviors remains unclear. In particular, anterior cingulate cortex (ACC) is said to motivate the successful completion of hierarchically-organized tasks by grouping together low-level actions according to superordinate goals, which are encoded across ACC neural ensembles as distributed, multivariate representations (Holroyd & Verguts, 2021; Holroyd & Yeung, 2012). Here we applied model-based representational similarity analysis (RSA) to investigate how rewards modulate these representations. We adapted an existing recurrent neural network (RNN) model of ACC activity (Colin et al., 2024) to predict the effect of rewards on ACC representations in a hierarchical sequence production task, and then recorded the fMRI-BOLD response from participants engaged in this task. As predicted, searchlight RSA revealed a cluster of MRI activity in ACC showing the highest second-order similarity with the model representation, relative to the whole-brain distribution. Closer examination revealed that ACC discriminates between rewarded vs non-rewarded sequences mainly on the first step of each trial, consistent with evidence that ACC encodes a reward prediction error at the onset of hierarchically organized action sequences. Moreover, reward-related multivariate ACC activity positively correlated with task performance, with more distinct ACC activity patterns predicting higher accuracy and faster response times. These findings were unique to ACC as other brain areas exhibited different task-related effects. We conclude that ACC monitors reward contingencies to motivate control over hierarchically-organized, goal-directed behaviors.

## Highlights

• ACC shows highest second-order similarity relative to the whole-brain distribution.
• Rewards modulate ACC representations mainly at step 1 of hierarchical sequences.
• More distinct ACC patterns correlate with improved task performance.
• Motivational signals refining ACC representations to improve task performance.

## 1 Introduction

The anterior cingulate cortex (ACC) is associated with a variety of cognitive processes including decision-making (Shenhav et al., 2014), conflict monitoring (Botvinick et al., 2001; Vanveen and Carter, 2002) and emotion regulation (Stuhrmann et al., 2011), but its specific function remains a topic of vigorous debate (Ebitz and Hayden, 2016; Silvetti et al., 2014). Despite this disagreement, several studies have highlighted an important role for ACC in the abilities to predict upcoming events in the environment and to allocate effort to task execution (Vassena et al., 2017). Early event-related potential (ERP) studies suggested that ACC produced reward (or ‘signed’) prediction error (RPE) signals indicating whether ongoing events are better or worse than expected, which were argued to reflect a contribution by ACC to value-based action selection (Holroyd and Coles, 2002; Holroyd and Umemoto, 2016); consistent with this hypothesis, recent evidence from intracranial recording studies with epilepsy patients (Oerlemans et al., 2025) and ERP studies with stroke patients (Oerlemans et al., 2024) have confirmed that these reward signals are produced in Broadmann Areas 24c’ and 32′ of ACC. Relatedly, other research has suggested that ACC generates *unsigned* prediction errors to unexpected events rather than to rewards in particular (e.g. Alexander and Brown, 2011; Shahnazian and Holroyd, 2018; Silvetti et al., 2011; see also Holland and Schiffino, 2016).

Central to this latter proposal is the idea that ACC encodes detailed representations (or “models”) of the environment that are monitored for unexpected events (Holroyd and Verguts, 2021). Although the term “representation” is interpreted differently throughout the subfields of psychology and neuroscience (Favela and Machery, 2023), here we refer to the collective activity of populations of interconnected neurons (Urai et al., 2022) and the transformations that occur to this information when transferred between neural systems (Barack and Krakauer, 2021; Kriegeskorte and Diedrichsen, 2019). This process can be simulated using artificial neural networks (ANN), which encode task functions across distributed sets of units in a brain-like manner (Rogers and McClelland, 2014; Schmidhuber, 2015). As described below, in previous work we found that information encoded by simple recurrent ANNs (RNNs) about task states (such as sequence position, goal identity, and so on) correspond closely to the distributed patterns of activity and prediction errors observed in ACC (Holroyd et al., 2018; Shahnazian and Holroyd, 2018).

With respect to effortful control of behavior, ACC activity is commonly seen in human fMRI studies to reflect allocation of cognitive resources. For example, Krebs et al. (2012) observed an interaction of the fMRI blood-oxygen-level-dependent (BOLD) signal in ACC between reward size and task difficulty, suggesting that ACC allocates cognitive resources for challenging tasks that offer substantial rewards. Subsequent research revealed increased ACC activity preceding more difficult or effortful tasks, irrespective of error, conflict, and choice (Vassena et al., 2014). In particular, participants exhibited concurrent activation in ACC and striatum when anticipating mentally demanding tasks with potentially higher rewards. This observation suggests that ACC estimates the effort required to obtain a reward, integrates this predicted cost with the expected reward into a net value signal, and applies the net value to motivate task performance (Vassena et al., 2014; see also Holroyd and McClure, 2015; Shenhav et al., 2013).

Although there is strong evidence that ACC contributes to reward processing and effort evaluation, the relationship of these functions to its concurrent role in encoding detailed representations of the environment are less clear. One clue stems from research suggesting that ACC motivates the execution of hierarchically high-level, abstract behaviors (Holroyd and Yeung, 2012). This perspective extends previous arguments about the neural-organization of complex human behavior, which posit that low-level actions are grouped according to hierarchically high-order goals mediated by prefrontal cortex (Botvinick et al., 2009). In particular, the hierarchical reinforcement learning (HRL) theory of ACC holds that ACC learns task values according to principles of reinforcement learning (RL) and selects tasks for execution based on these learned values (Holroyd and McClure, 2015; Holroyd and Yeung, 2012). Further, ACC motivates the execution of these tasks by directing other brain systems, such as the dorsolateral prefrontal cortex (DLFPC) and the basal ganglia, to execute the task sequence as a whole. In this way, ACC function is concerned with task selection and maintenance rather than with the details of action execution (Holroyd and Yeung, 2012).

To formalize this idea, we previously used RNNs to simulate how ACC encodes task progress during the production of goal-directed action sequences (Shahnazian and Holroyd, 2018). We then tested the predictions of this model using model-based representational similarity analysis (RSA) (Freund et al., 2021; Kriegeskorte et al., 2008) in an fMRI experiment in which subjects performed a “Coffee-Tea task” that required executing sequences of actions related to preparing coffee and tea (Holroyd et al., 2018). RSA evaluates the degree of second-order similarity between the representations encoded by different systems (Kriegeskorte et al., 2008). In our study, we applied multivariate techniques with RSA to compare the similarity structure of representations across task conditions within the RNN with the similarity structure of the representations across the same task conditions encoded in various brain areas. In particular, we constructed representational dissimilarity matrices (RDMs) from the model’s hidden unit activations and compared them to fMRI activation patterns using a whole-brain searchlight approach. This approach revealed brain regions with multivoxel response patterns corresponding to the model’s internal representational geometry, independent of the overall activation level. Consistent with the predictions of the RNN model, we found that human ACC encoded the largest cluster of brain activity showing high second-order similarity with the model representations (Holroyd et al., 2018). These representations were sensitive to information about both the stage and identity of each sequence relevant to the task context, indicating high sensitivity by ACC to distributed, dynamically evolving representations of extended action sequences. Note that these multivariate ACC representations were more finely grained than the larger-scale regional differences observed in other studies, including agent-specific spatial eigenmodes identified in self-referential contexts (Chiu et al., 2008) and subregional specialization associated with stress and affective demands (Gianaros and Wager, 2015).

Nevertheless, although the Coffee-Tea task is implicitly hierarchical, the RNN model used in the above study to simulate task performance contained no explicit hierarchy nor any means to exert cognitive control. For this reason, in a recent study we incorporated into the model goal units that facilitated the production of specific action sequences (Colin et al., 2024). These units provided top-down input that modulated the internal dynamics of the network, enabling it to stabilize selective representations associated with a given goal. By providing a means for the cognitive control system to bias the representations underlying specific hierarchical action sequences, the goal units served to make within-goal representations more similar relative to increased dissimilarity between across-goal representations. These simulations illustrate how cognitive control can regulate the dynamically evolving representations of task-related events encoded by ACC during the execution of hierarchically-organized action sequences.

In short, although ACC is a nexus for fundamental processes supporting reward evaluation, effort production, model-based prediction and the generation of hierarchically-organized behaviors, how ACC integrates these functions into an underlying representation remains unknown. Here we simulated the effects of reward on task-related representations in ACC by modulating the activations of the corresponding goal units in the hierarchical RNN model of the Coffee-Tea task (Colin et al., 2024). We then recorded the fMRI-BOLD signal in a modified version of the Coffee Tea task by rewarding performance on one of the two tasks (either coffee or tea, counterbalanced across participants). We investigated several predictions. First, we predicted that model-based RSA would reveal higher second-order similarity with ACC than with other brain regions (Colin et al., 2024; Holroyd et al., 2018). Second, in line with our previous simulations (Colin et al., 2024), we predicted that reward would influence the multivariate activity patterns in ACC by applying greater control over the rewarded sequences. As such, we expected that the task representations in ACC would be more dissimilar between the rewarded action sequences than between the non-rewarded action sequences. Third, we investigated whether these reward-related differences in ACC representations correlated with reward-induced improvements in task performance. Last, for comparison, we explored whether other brain regions exhibited reward-related effects differently from the effects observed in ACC.

To foreshadow our results, as predicted, ACC showed the highest correspondence between model-based and neural representations relative to the whole-brain distribution. Surprisingly, further analysis revealed that this region distinguished rewarded from non-rewarded sequences primarily at the first step of each trial. The distinctiveness of the ACC representations also correlated positively with task performance, with greater differentiation predicting higher accuracy and faster RTs. These findings suggest that reward information enhances task performance in part by modulating ACC representations of hierarchical task structure, particularly during the initial step of goal-directed action sequences.

## 2 Materials & methods

### 2.1 Data collection

#### 2.1.1 Participants

Fifty right-handed subjects (40 females, mean age: 22.5 years, range 18–39 years) with normal or corrected-to-normal vision participated in the study. None of the participants had a history of neurological or psychiatric conditions. The study was approved by the ethics committee of the HIRUZ Clinical Trial Unit (CTU) of Ghent University, and the experiment was conducted at the Ghent Institute for Functional and Metabolic Imaging (GIfMI) of Ghent University Hospital. Each participant signed an informed consent and a pre-scan list before the experiment. Subjects received 37 € for 1.5 h in the MRI scanner and an extra monetary bonus (mean bonus: 34.2 €, range: 29–36 €) based on their performance.

#### 2.1.2 Task

**Fig. 1:**
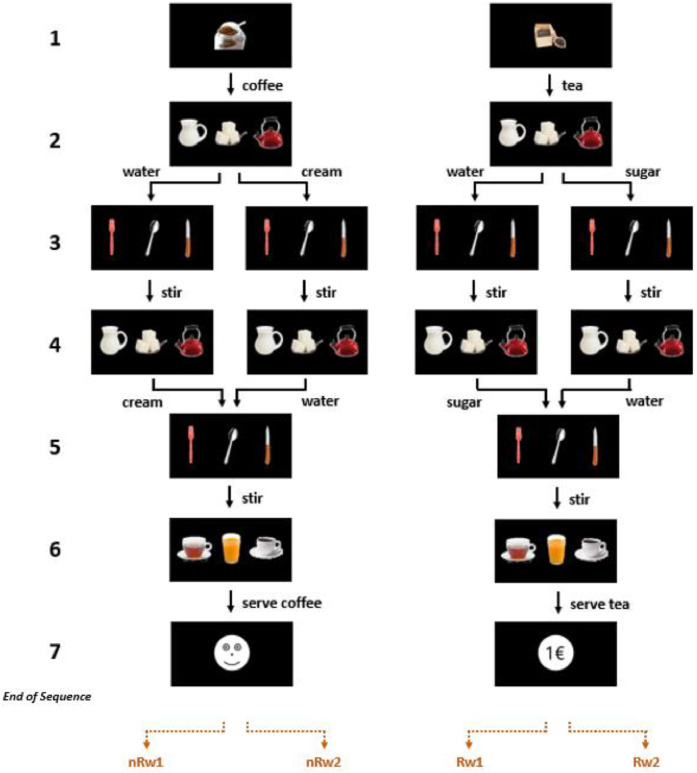
***The Reward-related coffee-tea making task.*** Each trial consisted of 7 steps (indicated on the left side of the figure). Step 1 was the cue step, signaling participants which task they need to perform, followed by 5 decision steps of 3-action choices each (step 2: cream, sugar, water; step 3: fork, spoon, knife; step 4: sugar, water, cream; step 5: spoon, knife, fork; step 6: prepared coffee, prepared tea, orange juice). For each decision, subjects selected one of the three actions by pressing a spatially corresponding button. Step 7 was the feedback step. Coffee or tea was rewarded, counterbalanced across participants (tea is rewarded in current figure for illustration purposes). Application of the task rules resulted in execution of four different task sequences (Rw1, Rw2, nRw1, nRw2). Rw1 & Rw2 indicates Rewarded trial with water as the first or the second ingredient, respectively. nRw1 & nRw2 refers to Non-rewarded trials.

Participants performed a “Reward-related coffee-tea making" (RCT) task, which is a modified version of a simple "coffee-tea making" task that was adapted for the purpose of fMRI (Holroyd et al., 2018). from a complex "activities of daily living" behavioral task (Schwartz et al., 1998). At the start of the experiment, the participants were asked to imagine that they were baristas at a coffee shop where they prepared cups of coffee and tea for their customers. Further, half of the participants were instructed that each successful preparation of a cup of coffee would earn them a 1 € tip, and the other half of the participants were instructed that each successful preparation of a cup of tea would earn them a 1 € tip, the order of which was counterbalanced across participants. For both groups, tips could not be earned for preparing the other drink irrespective of whether they were made correctly or incorrectly. The RCT task is a hierarchical sequence task consisting of 7 consecutive steps. During the first step of the task, participants were cued to make a cup of coffee or tea (Fig. 1, step 1). Then, a sequence of five decision steps followed, each characterized by a 3-item image describing three possible action choices (Fig. 1, steps 2–6). For each step, the same three items were displayed for all trials, but the items’ locations varied randomly from trial to trial. During each step, participants were required to choose one of the three options by pressing a spatially corresponding button. Each trial ended with a feedback stimulus indicating the correctness or incorrectness of the sequence executed on that trial (Fig. 1, step 7). In line with the instructions, one of the two tasks was rewarded for correct performance and the other task was never rewarded. At the end of each correctly-performed trial, participants received feedback in the form of a smiley face or a Euro symbol for the Non-rewarded and Rewarded conditions, respectively. For a wrongly-performed trial, a sad face or a 0€ sign was presented for the Non-rewarded and Rewarded conditions, respectively.

At the start of the experiment, participants were instructed to follow a set of rules to prepare coffee and tea successfully. First, cream can only be added to coffee, and sugar can only be added to tea. Second, both drinks require water. Thus, during steps 2 and 4 (see Fig. 1), participants were required to choose cream and water when making coffee and sugar and water when making tea. Third, the first of the two allowable ingredients on step 2 must be chosen randomly, “as if flipping a coin”. Fourth, the same ingredient cannot be added twice on the same trial. Thus, during step 4 the participants were required to choose the complementary ingredient to what was selected during step 2. For example, if they chose cream as the first ingredient when making coffee, then they were required to select water as the second ingredient during step 4. Fifth, for steps 3 and 5, the participants should always select the spoon (to stir the ingredient added on the previous step). Sixth, during step 6 the participants were required to select the drink that they prepared (in order to deliver it to their customer). Note that the images of the knife, fork, and orange juice were presented as distractors that the participants were required to ignore. If the drink was prepared correctly, then the participants were shown an image of a 1 € coin if the drink was rewarded or an image of a happy face if the drink was not rewarded. If the drink was prepared incorrectly, then they were shown an image of a 0 € coin if the drink was rewarded or an image of a sad face if the drink was not rewarded. They were told that the feedback signified the presence or absence of a tip (on the rewarded trials) and a happy or sad customer (on the non-rewarded trials). Participants were explicitly informed that they would receive the full bonus subsequent to the task completion (mean bonus: 34.2 €).

Successful application of the above rules resulted in four unique action sequences, for example, execution of a non-rewarded coffee or tea sequence with water as the first ingredient (i.e., coffee or tea – water – stir – cream or sugar – stir – coffee or tea; see Fig. 1, left-most pathway, which shows an example of a non-rewarded coffee with water as the first ingredient). Note that the images for steps 2–6 were identical for all four sequences. Thus, participants needed to maintain contextual information throughout each trial about whether they were making coffee or tea and which ingredient they added first. This combination of six steps and four possible action sequences resulted in 24 different task states.

#### 2.1.3 Computational model

**Fig. 2:**
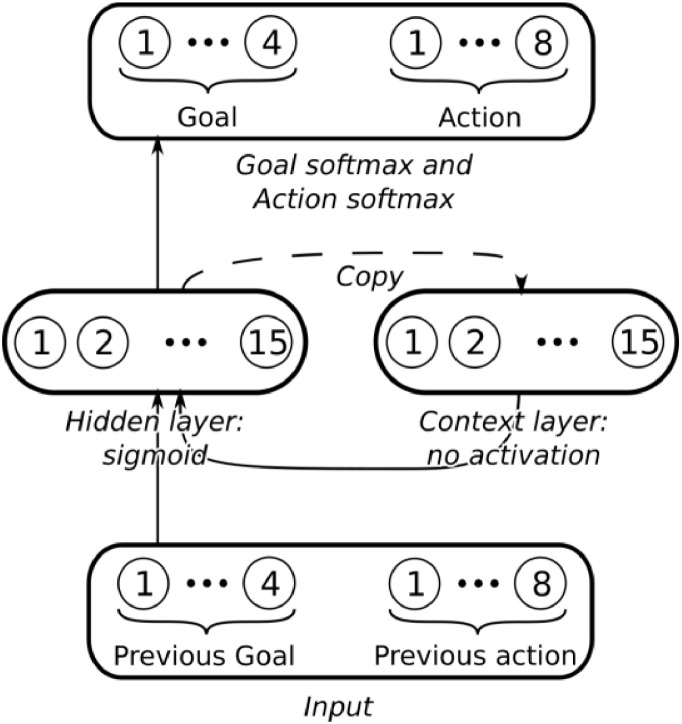
***4 goal-units recurrent neural network (RNN) architecture (adapted by****Colin* et al.*, 2024****).*** The model simulates reward effects on ACC task representations of the four action sequences. It includes three layers: an input layer with 8 units for previous actions or initial cues, a recurrent hidden layer with 15 units, and an output layer with 8 units for target actions. Four goal units correspond to the possible sequences. The network was trained using backpropagation through time. Post-training, no further learning occurred; at test time, goal unit activations were modulated to simulate Rewarded vs. Non-rewarded sequences. Hidden layer activations were recorded to analyze the effect of rewards on ACC representations.

We applied an existing recurrent neural network model of ACC (Colin et al., 2024) to simulate the effect of rewards on ACC representations of the four action sequences in the RCT-task (see also Holroyd et al., 2018). The network consisted of an input layer, a hidden layer with recurrent connections, and an output layer (Fig. 2). The input layer included 8 units corresponding to the previous action ("add coffee", "add tea", "add milk", "add water", "stir", "add sugar") or the tea/coffee cue (“make tea”, “make coffee”) shown to participants at the beginning of each sequence (step 1). The hidden layer consisted of 15 units with sigmoid activation, with recurrence in the manner of Elman (1990), (Holroyd et al., 2018): the network’s hidden layer activations were saved at each time-step to a separate context layer, which served to provide fully-connected input to that same layer on the subsequent time step. Finally, the output layer consisted of 8 units, corresponding to the 8 possible target actions ("add coffee", "add tea", "add milk", "add water", "stir", "add sugar", "serve coffee", and "serve tea"), with softmax activation. Additionally, the network incorporated 4 goal units with softmax activation corresponding to each of the four possible sequences, which served both as inputs to the hidden layer and output from it.

The weights were initialized to small values sampled from a normal distribution (mean 0 and standard deviation 0.1). The loss was defined as the sum of the cross-entropy losses for the target goals and actions. The network was trained by backpropagation through time (BPTT) using stochastic gradient descent. On the first step of each sequence, the inputs consisted of the goal unit corresponding to that sequence, and either “make tea” or “make coffee”; context layer activations were set to zero. 25 networks were trained from independent weight initializations; after training for 5000 episodes, all network instances could perform the task with full accuracy (Suppl. Table 1). Note that the goal of training the networks was not to model how people learn the coffee-tea task, which undoubtedly depends on a different learning mechanism. Rather, the networks were trained in order to bring the system parameters into a range capable of the network emulating human cognitive functions, as has been the practice in numerous studies in cognitive psychology (e.g., Servan-Schreiber et al., 1991; Botvinick and Plaut, 2004).

**Table 1:** ***Three-way repeated measures ANOVA results on step distances in ACC****.* A three-way repeated-measures ANOVA was conducted on the distances for each step of the four task sequences, with Reward condition (Rewarded vs. Non-rewarded), Step (1 to 6), and Task (coffee vs. tea) as factors.

Anterior Cingulate Cortex					
	df	Sum of Squares	Mean of Squares	F-value	p_value_
**Condition**	**1**	**2.83E+08**	**2.83E+08**	**5.914**	0.0151 *
**Step**	**35**	**4.43E+10**	**1.27E+09**	**26.428**	<2E-16 ***
Task	1	1.65E+05	1.65E+05	0.003	0.9532
Condition * Step	**35**	**2.67E+09**	**7.64E+07**	**1.593**	0.0149 *
Step * Task	35	9.07E+08	2.59E+07	0.541	0.9876
Condition * Step * Task	35	6.46E+08	1.85E+07	0.385	0.9996
Residuals	3408	1.63E+11	4.79E+07		

After training, the network weights were frozen and the goal units were used to simulate rewarded vs. non-rewarded sequences. Specifically, non-rewarded and rewarded action sequences were simulated by fixing the corresponding goal unit activation at 0 or 1, respectively, during the execution of the sequence. Importantly, no further learning occurred after training; the reward manipulation was implemented solely by adjusting goal unit activation at test time. We found that networks averaged approximately 80 % of correct actions on non-rewarded sequences compared to 100 % on rewarded sequences, in line with the effects of rewards on human behavior (i.e., better performance on the rewarded tasks; see Section 3.1). The activations of the hidden layer were recorded for each step of each sequence, and the Euclidian distances between the activation vectors of the hidden layer for all pairwise combinations of states were computed, separately for two types of representational dissimilarity matrices (RDMs) corresponding to (1) rewarding only the coffee sequences, and (2) rewarding only the tea sequences. To ensure reliability of these RDMs, we used the average distances for the 50 independently trained networks.

We anticipated that the context layer of the RNN would represent several task characteristics, including sensitivity to the contextual information essential to the successful execution of the 4 action sequences (Holroyd et al., 2018). Furthermore, the inclusion of goal units was intended to simulate the effect of reward on these contextual representations. Crucially, we expected that representations in the context layer of the artificial neural networks would correlate more strongly with ACC representations than with the representations of other brain regions.

#### 2.1.4 Experimental procedure

The task was coded on Expyriment (version 0.9.0, running on Python 3.8), a python library for cognitive and neuroscientific experiments (Krause and Lindemann, 2014). Each step was presented for 1 s and was followed by a black screen for 2.5–4.5 s (mean 3.5 s, with 7 possible jitters at 0.33 s increments). If a response was not recorded during the 1 s stimulus presentation or during the following black screen, a “Too Late” message appeared on screen for both Rewarded and Non-rewarded conditions.

Before starting the fMRI session, participants received detailed instructions about the task and performed six training trials of making coffees and teas without monetary rewards. At the end of the training, they were informed that inside the scanner, they would earn 1 € for each successful preparation of either coffee or tea (but not both), counterbalanced across participants. In the scanner, they performed four blocks of the task, each consisting of 18 trials. This resulted in 72 trials in total, including 36 Rewarded trials and 36 Non-rewarded trials. At the end of each block, participants were given a self-paced break. To facilitate an even selection of task sequences, during the break they were also presented with feedback indicating the number of coffees and teas with water as the first ingredient that they produced, and a reminder to prepare drinks with either cream/sugar or water as the first ingredient about equally often. After exiting the scanner, participants responded to a set of questions about their task strategies (if any), completed the Behavioral Inhibition, Behavioral Activation and Affective Responses (BIS/BAS) questionnaire, which assess individual differences in reward sensitivity (Carver and White, 1994), and were debriefed.

#### 2.1.5 Functional magnetic resonance imaging data acquisition

Structural and functional images were acquired using a Siemens 3 T Magnetom Trio MRI scanner equipped with a 64-channel radiofrequency head coil. At the beginning of the scanning session, an anatomical, T1-weighted structural image was acquired (MPRAGE pulse sequence, TR=2250; TE=4.18 ms; 176 slices; voxel size = 1 mm x 1 mm x 1 mm; FoV=256 mm). A gradient-echo fieldmap was used to correct for distortions due to use of a multi-band sequence. Functional images were acquired with an echo-planar imaging (EPI) pulse sequence (52 slices per volume; TR=1720 ms; TE=27 ms; slice thickness = 2.5 mm; voxel size = 2.5 mm × 2.5 mm × 2.5 mm; FoV=210 mm; flip angle =66°). During each run, we collected 362 vol and the task was synchronized to begin at the sixth volume; the first five volumes were discarded during the analyses.

### 2.2 Statistical analysis

In all analyses, except for the calculation of accuracy, only data from trials with correct responses were included (i.e., excluding late responses and errors). Error trials were not included in our analyses because they were infrequent and unevenly distributed across conditions and blocks, limiting their interpretability. Analyses of the behavioral data were carried out in the R environment for statistical computing and visualization (R version 4.3.0) and the fMRI analyses were conducted using custom-made scripts on Matlab R2021b and SPM12 software (Statistical Parametric Mapping; Wellcome Department of Imaging Neuroscience, London, UK). For the multivariate analyses, the Decoding Toolbox (TDT; Hebart et al., 2015) was used for the classification analyses and the RSA Toolbox (Nili et al., 2014) for the RSA.

#### 2.2.1 Behavior

Within-subjects mean response times (RTs) and accuracies were computed using the Rmisc package (Hope, 2012). To combine RT and accuracy into a single performance metric, we calculated the Balanced Integration Score (BIS) (Liesefeld et al., 2015). The BIS is defined as the difference between standardized accuracy and standardized RT, ensuring both measures are on the same scale and equally weighted. Positive BIS values indicate better performance (faster RTs and higher accuracy), while negative values reflect worse performance (slower RTs and/or lower accuracy).

BIS scores were calculated separately for the rewarded and non-rewarded conditions for each participant. We then computed the contrast between these two BIS scores (rewarded minus non-rewarded), with positive difference values indicating overall better performance for rewarded sequences compared to non-rewarded sequences.

#### 2.2.2 Neuroimaging

The functional images were pre-processed and analyzed using SPM12 software in Matlab (Wellcome Trust Centre for Neuroimaging). The pre-processing was performed in six steps. First the T1 images were defaced to anonymize the data and the fieldmap was calculated. Afterwards, the images were re-aligned to the first image in each time series and unwrapped to correct for movement artifacts and magnetic field inhomogeneities, using the calculated fieldmap. Then, the images were corrected for slice time acquisition order, co-registered to the mean EPI image and segmented. Finally, the images were spatially normalized to MNI space for the purposes of univariate analysis. For the multivariate analysis, data were not normalized nor smoothed to preserve fine-grained patterns of voxel activations (Haynes and Rees, 2006).

The pre-processed data were used to estimate a voxel-wise general linear model (GLM). The GLM included a separate regressor of interest for each of the 24 task states, resulting in 24 regressors, and six nuisance regressors for motion parameters. Task regressors were time locked at the onset of each step with a duration of 1 s and convolved with a canonical hemodynamic response function as implemented by SPM. Automatic Anatomic Labelling atlas (Rolls et al., 2020) was applied to all the images.

#### 2.2.3 Searchlight representational similarity analysis

Searchlight RSA was employed to test our main prediction that the model-based RDM would show stronger second-order similarities with representations in ACC than with other brain regions. RSA assesses the correspondence between second-order isomorphisms associated with qualitatively different sources (Kriegeskorte et al., 2008). Following the example of our previous studies (Colin et al., 2024; Holroyd et al., 2018), these sources are the RNN predictions and the fMRI-BOLD data. RSA was used to measure the dissimilarity of task action sequences by building an RDM following the same format for the RNN model (see Section 2.1.3). In particular, the “brain” RDM was built by computing a 24 × 24 distance matrix constructed from the 4 sequences of 6 steps each. To conduct the searchlight RSA a vector of BOLD signal activations was derived from each 6 mm radius sphere surrounding each voxel in the volume, for each of the 24 task states. This resulted in 24 vectors for each voxel, reflecting the pattern of neural activity in each volume of the 24 task states. This was done for each participant separately. For each voxel, a separate RDM was derived by computing the cross validated Euclidean distance across all pairwise combinations of the 24 states.^1^ Cross validation was performed using a leave-one-run-out approach, with distance values per cross-validation fold averaged to obtain the final dissimilarity values for each of the 24 × 24 searchlight RDMs. The upper triangle of these RDMs was vectorized (ignoring elements along the diagonal), yielding a 276- unit vector for each voxel in the volume. Likewise, the upper triangle of the model-RDM was vectorized (ignoring elements along the diagonal), yielding a 276-element vector. Fourth, for each subject, the Spearman’s rank correlation was computed between the 276-element model vector and each 276-element brain activation vector, yielding a correlation value for each voxel in the brain volume. To test the hypothesis that the representations in ACC correspond more strongly to the model-RDM relative to other brain regions, the correlation values were z-transformed within-subjects; larger values therefore indicate that the pattern of activation of a brain area correlates more strongly with the model-RDM relative to the whole-brain distribution of correlations within that subject (Holroyd et al., 2018). Finally, the results were spatially smoothed using a Gaussian kernel (6 mm full-width at half maximum). For each participant, the above analysis was performed using the RNN-RDM appropriate to whether the participant was rewarded for making coffee or tea. During the second-level group analysis, data of those two separate RSA analyses were then combined by applying one-sided signed-rank tests on the correlation values to identify which neural voxel-based searchlight RDMs were significantly correlated with the RNN-RDM across participants, thereby revealing which brain region representations correlated most strongly with the RNN representations. Increased probability of Type 1 errors due to multiple comparisons was mitigated with a familywise error (FWE) cluster correction of 0.05 and a primary voxel-wise threshold of *p_value_ < 0.001*.

#### 2.2.4 ROI classification analysis

To analyze further the representational structure of the clusters yielded by the RSA, we conducted an exploratory ROI cross-classification analysis using the clusters identified by the RSA as the ROIs. ROI cross-classification was run using TDT (Hebart et al., 2015) on the GLM parameter estimate maps of the 24 states for each individual subject. Data were classified using a cross validated Euclidean distance metric following a leave-one-run-out approach. Therefore, for each subject, every run was the test dataset once. This yielded a 24 × 24 RDM of the Euclidean distances of all pairwise combinations of the 24 task states per participant. From each participant’s 24 × 24 RDM, we selected two 6 × 6 subsets of distances to analyze representational differences related to reward. One subset includes the distances between the task states occurring within the rewarded sequences, and the other subset includes the distances between the task states occurring within the non-rewarded sequences (72 distances in total for each participant). Focusing the analysis on this subset of distances allowed for testing whether reward influences the structure of the representations across the steps in each sequence while controlling for step progression. A linear mixed effects model was fitted to the data using the *lme()* function from the *nlme* package in R (Pinheiro et al., 1999), with distances as the dependent variable and reward condition, step progression and task, and all of their possible interactions, as the independent variables. Individual subjects were treated as random effects to account for the nested structure of the data. Post-hoc pairwise comparisons on the interaction between reward and step progression were performed using the *emmeans()* function (Lenth, 2024). For the purpose of illustration, nonmetric multidimensional scaling (MDS) was applied to the cross-validated Euclidean distances, separately for each ROI, using the MATLAB command:

$$\;\text{yy}=\text{mdscale}(1-\text{cc},2{,}^{\prime }{\text{start}}^{\prime }{,}^{\prime }{\text{random}}^{\prime }{,}^{\prime }{\text{criterion}}^{\prime }{,}^{\prime }{\text{metricsstres}}^{\prime });.\;$$

## 3 Results

### 3.1 Behavior

**Fig. 3:**
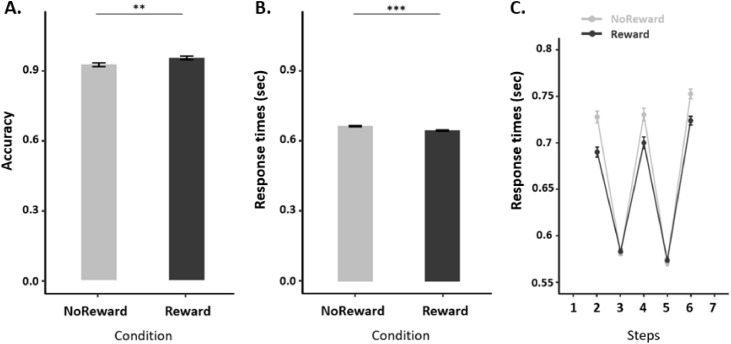
***Behavioral Results.*** (A) Accuracy, (B) Response Times (RTs) and (C) RTs across the sequence steps (1–6) for Rewarded and Non-rewarded conditions. Reward significantly improved the behavioral performance of the participants. Error bars indicate within-subjects standard error of the mean (SEM). Significant differences: **p < 0.01, ***p < 0.001.

A within-subjects *t*-test on accuracy between the Reward and the Non-reward conditions indicated that the participants were significantly more accurate on the Reward trials (*M* = 0.95, SD = 0.054) than on the Non-reward trials (*M* = 0.92, SD = 0.05), t(49) = −2.71, *p = 0.01* (Fig. 3A). A two-way ANOVA on the RTs of each step (Steps 2–6) and the reward condition (Reward, Non-reward) revealed a significant main effect of reward, such that participants were faster on Reward trials (*M* = 0.65 ms, SD = 0.28 ms) than on Non-reward trials (*M* = 0.67 ms, SD = 0.31 ms), F(1, 16,825) = 29.49, *p <* *0.001* (Fig. 3B). Further, there was a significant main effect of step (F(4,16,825) = 383.90, *p* *<* 0*.001*) and a significant effect of the interaction between reward and step (F(4,16,825) = 5.52, *p* *<* 0*.001*). Post hoc testing using Tukey’s HSD indicate that participants were significantly faster on Reward trials for steps 2,4 and 6 than on Non-reward trials (*p_2_ <0.001, p_4_ <0.01, p_6_ <0.01* for steps 2, 4 and 6, respectively) (Fig. 3C). Note that these results indicate that the rewards induced participants to be both more accurate and faster on the rewarded trials, rather than to trade-off accuracy against speed.

To confirm this inference, BIS scores were computed separately for the rewarded and non-rewarded conditions for each participant and then the contrast between these scores was calculated; positive values indicate overall better performance (faster RTs and higher accuracy) for rewarded sequences compared to non-rewarded sequences. A one-sample *t*-test confirmed that the mean BIS score (0.52 ± 1.1) was significantly greater than zero, (t(49) = 3.36, *p* = 0.0015), indicating that rewards were associated with better task performance overall. To further investigate the relation between the BIS contrast and task performance metrics, we computed the *RT contrast* and the *accuracy contrast* for each participant, defined as the difference between these values for the two conditions (rewarded minus non-rewarded). These contrasts were then correlated with the BIS score contrasts in order to elucidate the relative dependence of the BIS scores on RTs vs. accuracies. BIS contrasts were strongly positively correlated with the accuracy contrast (*t* = 8.81, *p* < 0.001) and negatively correlated with the RT contrast (*t*=−4.55, *p* < 0.001) (Table 2), indicating that the rewards primarily enhances performance by improving accuracy while also speeding up the RTs.

**Table 2:** Pearson Correlations between BIS Score Contrast, Task Performance Contrasts, and Neural Activity contrasts. This table presents Pearson correlation coefficients between the BIS score contrast and task performance contrasts (RT contrast and accuracy contrast), as well as neural activity contrasts (distances contrast and beta values contrast) in caudal ACC. Each contrast is defined as the difference between the rewarded and non-rewarded condition for the associated measure.

Correlation Table						
				Pearson		
				t	r	p
BIS contrast	–	RT contrast		−4.55	−0.55	<0.001
BIS contrast	–	Accuracy contrast		8.81	0.79	<0.001
BIS contrast	–	Distance contrast ACC	Steps 1–6	2.95	0.39	<0.01
BIS contrast	–	Distance contrast ACC	Step 1	5.68	0.63	<0.001
BIS contrast	–	Beta values contrast ACC	Steps 1–6	3.00	0.40	<0.01
BIS contrast	–	Best values contrast ACC	Step 1	4.09	0.51	<0.001

### 3.2 Model predictions of reward modulation of hierarchically-organized representations

Our main hypothesis concerned how reward modulates the encoding by ACC of distributed representations of hierarchically-organized, goal-directed action sequences. To investigate this question, we used an RNN model that was originally used to capture task representations in ACC (Holroyd et al., 2018) and that was later modified to incorporate goal units that bias the activity of corresponding task sequences (Colin et al., 2024). In particular, we assumed that rewarding particular action sequences would enhance top-down regulation over the execution of those sequences, which we simulated by boosting the activity of the corresponding goal units.

**Fig. 4:**
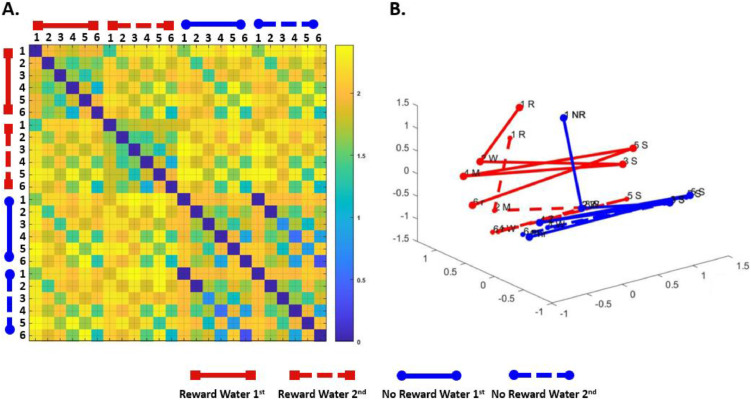
***RNN-based Model Predictions of rewarded sequences.*** (A) Representational dissimilarity matrix (RDM) displaying Euclidean distances between pairwise activation patters of the hidden layer for rewarded (in red) and non-rewarded (in blue) sequences. (B) Multidimensional scaling (MDS) plot illustrating the pairwise Euclidean distances in a reduced-dimensionality state space. The scale represents the magnitude of dissimilarity (arbitrary units), with larger values indicating greater differences in the hidden layer representations.

The model RDMs revealed distinct activity patterns that were differentially modulated by reward value across conditions. Specifically, in the rewarded condition, the RDM (Fig. 4A) and the associated MDS plot (Fig. 4B) indicate that reward increases the representational dissimilarity within a rewarded drink, making task states more distinct and introducing structure as compared to the non-rewarded condition (Fig. 4B red versus blue lines). Furthermore, within-subtask dissimilarities (e.g., rewarded sequence with water-first versus water-second) were greater in rewarded sequences, with more distinct and separated representations observed between subtasks of the two rewarded task sequences compared to the two non-rewarded sequences (Fig. 4B; note the clear divergence between the solid and dashed red lines, in contrast to the clustering of blue lines). Overall, the model predicts clear separability between the coffee and tea sequences when either one is rewarded, with rewarded sequences demonstrating more task-specific differentiation across subtasks.

### 3.3 Model-based RSA loads most strongly onto ACC

**Fig. 5:**
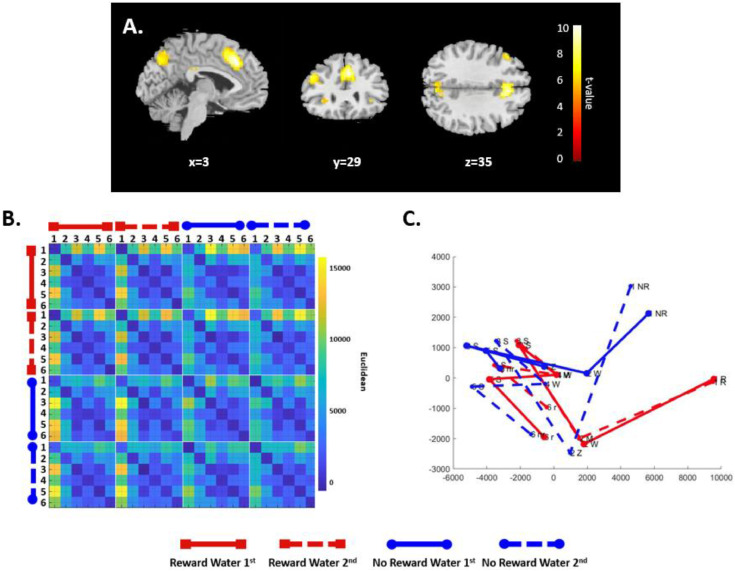
***Multivariate Analysis in caudal ACC.*** (A) Correlation map highlighting the largest cluster identified by the recurrent neural network (RNN) model representational dissimilarity matrix (RDM) - representational similarity analysis (RSA) in caudal ACC, consisting of 310 voxels (coordinates, *x* = 3, *y* = 29, *z* = 35). (B) RDM of Euclidean distances across all pairwise combinations of the task steps for Rewarded and Non-rewarded conditions (arbitrary units). (C) Multidimensional scaling (MDS) plot illustrating the dynamics of the ACC state transitions with reduced dimensionality (arbitrary units). The ACC encodes the reward-related coffee and tea making task more similarly to the RNN model than do other brain regions.

To investigate how reward modulates ACC encoding of distributed representations of hierarchically-organized, goal-directed action sequences, we applied a searchlight RSA to compare the model predictions to the fMRI-BOLD response collected in the RCT task. We predicted that the neural representations in ACC would show the highest second-order similarity of all brain areas with the patterns of activation encoded by the hidden units of the model. Consistent with this prediction, the most strongly correlated cluster in the searchlight RSA was in medial-frontal cortex centered on ACC (caudal ACC, 310 voxels, *x* = 3, *y* = 29, *z* = 35), with the peak t-value located in the right cingulate gyrus in Brodmann area (BA) 32 (Fig. 5A, Suppl. Table 2C). This cluster extended dorsally and posteriorly through caudal ACC and the presupplementary motor area (pre-SMA, including BA 8, 9 & 32) (Kim et al., 2010). These findings indicate that ACC encodes the RCT more similarly to the RNN model than most other brain regions do, as revealed by its position at the upper end of the whole-brain similarity distribution following z-transformation

We next examined the specific impact of the rewards on the ACC task representations. Toward this end, we performed an ROI classification analysis using TDT (Hebart et al., 2015) on the GLM parameter estimate maps of the ACC cluster derived from the searchlight RSA for every pairwise combination of the 24 states (Fig. 5A). As described in the methods, we constructed a 24 × 24 RDM for the 24 different task states using cross-validated Euclidean distance as distance metric and further inspected the results using MDS. These results suggest that reward has a particularly strong effect on the representations in ACC during step 1 of the trial (Fig. 5B-C). To investigate this observation, a three-way repeated measures ANOVA performed on the ACC distances with reward condition, step progression and task as factors revealed a significant main effect for reward F(1, 3408) = 5.91, *p = 0.015*, a significant main effect for step progression F(35, 3408) = 26.428, *p < 0.001* and a significant interaction between reward and step progression F(35, 3408) = 1.59, *p = 0.015*. There was no significant main effect for task F(1, 3408) = 0.003, *p* = 0.95, and all other interactions (reward – task; step progression – task; reward – task – step progression) were not significant (Table 1). Post-hoc analysis indicated that reward affected only the distances between step 1 and the steps 2, 3 and 5 of the rewarded sequences (Suppl. Table 3).

To explore how behavioral performance relates to neural activity in caudal ACC, we examined the relationship between the BIS score contrast (rewarded minus non-rewarded) and the distance contrast in caudal ACC, defined as the difference in multivariate distances between rewarded and non-rewarded conditions (Table 2). Across all steps, we observed a significant positive correlation between the BIS score contrast (indicating relatively better performance for rewarded sequences) and larger multivariate distances in caudal ACC (indicating greater neural differentiation between rewarded and non-rewarded task states) (*t* = 2.95, *p* < 0.01). This suggests that participants whose performance benefited more from reward showed more distinct neural representations in this brain region.

Given the strongest reward effects at step 1, we conducted a separate exploratory analysis for step 1, which yielded an even stronger correlation (*t* = 5.68, *p* < 0.001) (Table 2). For completeness, we also analyzed the relationship between the BIS score contrast and the beta value contrast, defined as the difference in beta values between rewarded and non-rewarded conditions, both for all steps and for step 1 separately. Note that whereas multivariate distances capture the distributed patterns of neural activity across this ACC cluster, the average of the beta values reflect the average activation within the region. Our analysis showed that the beta values, like the multivariate distances, exhibited a strong correlation with BIS scores, particularly at step 1. However, these effects were less pronounced than those seen in the multivariate analysis (Table 2), suggesting that while average neural activity increased with better behavioral performance, it is the distributed patterns of activity – reflected in the multivariate distances – that provides a more robust account of how caudal ACC supports task performance in the context of rewards. This suggests that the impact of rewards on task performance relates more closely to the neural representations in ACC rather than simply to the overall magnitude of the activation.

Lastly, we administered BIS/BAS scales (Carver and White, 1994) to all our participants, but found no significant correlations between its categories and behavior or multivariate distances within caudal ACC.

### 3.4 ACC plays a distinctive role during execution of hierarchically-organized action sequences

Although our primary focus was on ACC, we also examined the next three most significant clusters identified by the model-based RSA: the occipital gyrus, cerebellum, and precuneus. For each ROI, we applied the same multivariate classification and statistical analysis used for ACC. While all three clusters exhibited effects of reward, their representational profiles differed from ACC in meaningful ways, including greater sensitivity to task identity and reward effects that extended beyond the first step. Full results are reported in Supplementary Material, Section 2.

**Fig. 6:**
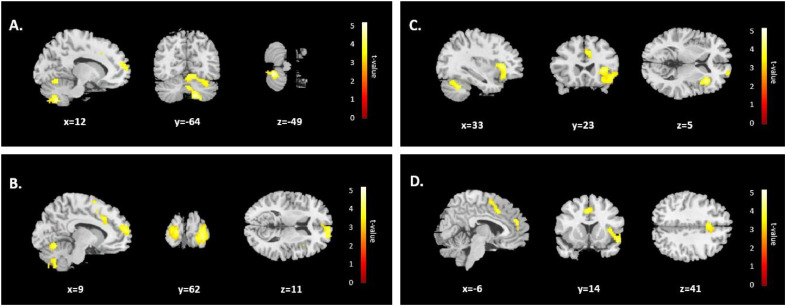
***Searchlight Classification Analysis on Reward* vs. *Non-reward.*** (A) Right cerebellar hemisphere. (B) Medial frontal gyrus (rostral ACC). (C) Right insula. (D) Middle cingulate gyrus (caudal ACC). For each region, searchlight classification analysis reveals significant activations distinguishing between Rewarded and Non-rewarded conditions. These activations indicate that reward-related differences in task representation extend beyond ACC to other brain areas involved in cognitive and motor control.

Finally, we explored whether other brain regions also show a main effect of reward by conducting a leave-one-run-out searchlight classification analysis that discriminated between rewarded versus non-rewarded sequences, allowing for a whole-brain examination of purely reward-related effects. This analysis yielded significant clusters of activations in the right cerebellar hemisphere (134 voxels), the medial and dorsolateral frontal gyrus (229 voxels), the lobule VI of vermis (278 voxels) and the right insula (274 voxels) (Fig. 6, Suppl. Table 2D), identifying a broader neural network that differentiates between rewarded and non-rewarded states.

## 4 Discussion

ACC supports an array of cognitive functions related to reward processing, effort evaluation, cognitive control, and the production of hierarchical action sequences, yet how it integrates these functions into an underlying representation remains unclear. The complex interplay between these processes highlights the important role of ACC in aligning motivational factors with cognitive demands in order to carry out goal-directed behavior. To investigate this interaction, the present study employed a hierarchical action sequence task in combination with predictive neural network modelling of the effects of cognitive control on goal-directed behaviors (Colin et al., 2024; Holroyd et al., 2018). Following the approach of our previous work (Holroyd et al., 2018). we simulated the effects of reward on ACC representations using an RNN model that incorporates goal units for this purpose (Colin et al., 2024). Modulating the activation levels of the goal units in accordance with the execution of rewarded vs. non-rewarded task sequences induced distinct patterns of activation across the network’s hidden units. These simulated representations were then compared using RSA with the fMRI activity of participants conducting the same task. As predicted, the model-based RSA exhibited the strongest loading on ACC compared to all brain areas, confirming the relatively robust correspondence between patterns of ACC activity and the model representations. It is important to note that this conclusion is based on within-subject z-transformed correlations, which reflect relative correspondence across the whole-brain distribution rather than absolute differences between regions.

To explore this result, a classification analysis on all pairwise combinations of the 24 states in this ACC cluster revealed a significant main effect of reward on the ACC representations, as expected. Perhaps surprisingly, this effect was strongest by far on step 1 of each trial (Fig. 5B, Fig. 5C), when the participants were informed whether they should make a cup of tea or a cup of coffee on that trial (Fig. 1). Tellingly, the peak value of this cluster closely aligns with the source of a reward-related ERP component called the reward positivity (Holroyd and Umemoto, 2016; Cavanagh and Holroyd, 2025), as revealed by previous studies that investigated this question using fMRI (Stringfellow et al., 2024), intracranial EEG (Oerlemans et al., 2025), and the consequences of stroke damage on the ERP (Oerlemans et al., 2024). Three decades of research has indicated that the reward positivity reflects a (signed) RPE signal that is larger to unexpected positive events than to unexpected negative events (Sambrook and Goslin, 2015), possibly produced by RPE signals carried to ACC by the midbrain dopamine system (Holroyd and Coles, 2002). Evaluated in this context, we suggest that the strong reward-related signal elicited at the beginning of the rewarded hierarchical action sequence reflects a positive RPE signal used by ACC to motivate successful execution of the subsequent sequence of actions (Holroyd and McClure, 2015; Holroyd and Yeung, 2012; Rushworth et al., 2004; Shenhav et al., 2013).

Consistent with this inference, the BIS score contrast between the rewarded and the non-rewarded sequences was positively correlated with the multivariate distances contrast in caudal ACC between the rewarded and the non-rewarded condition, particularly during the first step when the reward response was largest. This indicates that greater neural differentiation in caudal ACC supports enhanced task performance in rewarded contexts by modulating the distinctiveness of neural representations. The finding aligns with observations by Krebs et al. (2012) and Aarts et al. (2008) that highlight the role of ACC activation in reward processing and cognitive control, but goes beyond these previous findings by demonstrating that rewards do so by influencing the distinctiveness of multivariate neural representations underlying task sequences. These observations highlight the dual role of ACC in integrating motivational signals with task-relevant representations.

Note that the model was trained to about 100 % accuracy across all of the sequences. However, when simulating the task proper, the reduction of the goal unit activation on the non-rewarded trials caused performance on those trials to drop to about 80 % accuracy. This manipulation was intended to simulate reduced motivational control over these sequences. Because the model was trained before the task was carried out by the participants, we did not fit the model parameters directly to human behavioral data. Hence the model performed somewhat worse than the human participants did on these Non-rewarded trials (which ranged from 41 %−100 %, *M* = 92 %), but this drop in performance matched the empirical data in a qualitative way.

Although we were mainly concerned with the effects of reward on ACC representations of hierarchical tasks, we explored whether these effects were unique to ACC by applying the same analysis to other brain regions also identified by the model-based RSA: the occipital gyrus, precuneus and posterior cerebellum (Suppl. Material, Section 2). Notably, like the ACC, all three brain areas exhibited effects of reward. However, the representations of these brain areas also deviated from the ACC representations in ways leading to overall weaker loadings of the RNN model RDM, resulting in smaller cluster sizes. First, posterior cerebellum showed main effects of both reward and step progression, but no interaction between reward and step (Suppl. Table 4B), indicating that the influence of reward on the task representations extends throughout task execution (not just the first step). The cerebellum plays a critical role in both motor and cognitive task sequencing, ensuring that actions are performed in the correct order and with proper timing (Stoodley and Schmahmann, 2010). Reward feedback might thereby enhance the cerebellar contribution to task execution on rewarded trials, in particular by modulating task performance via its influence on the medial prefrontal cortex through the thalamus (Rogers et al., 2011). In this manner the prospect of reward could induce the cerebellum to optimize action sequence and timing across all of the steps of all of the sequences.

Second, in contrast to ACC, the representations of which were insensitive to differences between the coffee and tea tasks, the occipital gyrus and precuneus exhibited a significant main effect of task (Suppl. Table 4A & 4C). Occipital gyrus and precuneus are both involved in processing visual information and integrating sensory inputs. In the coffee-tea task, occipital gyrus could distinguish task-specific visual attributes via attentional mechanisms that focus on relevant task features (e.g., tea leaves versus coffee grounds) (Grill-Spector and Malach, 2004; Serences and Yantis, 2006). Similarly, precuneus integrates visual cues with cognitive functions and is involved in episodic memory (Cavanna and Trimble, 2006; Lundstrom et al., 2005), making it sensitive to the context and details of the coffee-tea task. By contrast, although we cannot conclude that ACC is completely insensitive to task differences based on the absence of a task effect, the relative sensitivity to reward value suggests that it operates at a more abstract, goal-oriented level that is concerned with the formal structure of the tasks, rather than with the specific details of task implementation. This aligns with the notion that ACC is involved in higher-level cognitive control and abstraction that guide behavior based on overarching goals or principles rather than detailed task-specific elements (Holroyd and Yeung, 2012). Such abstraction can facilitate contextual flexibility, allowing neural systems to generalize across different situations (e.g. Ostojic and Fusi, 2024).

This division of labor within the brain’s reward network highlights ACC’s role in integrating reward signals with hierarchical task representations. Our findings show that ACC selectively enhances the distinctiveness of neural representations during rewarded tasks, particularly at the initiation of goal-directed sequences, enabling precise and efficient task execution. This specificity contrasts with the task-detail sensitivity observed in regions like the cerebellum and occipital cortex, suggesting that ACC operates at a more abstract, goal-oriented level within the reward network.

## Ethics statement

This study was conducted in accordance with the principles outlined in the Declaration of Helsinki and the International Conference on Harmonization for Good Clinical Practice (ICH/GCP) guidelines. Ethical approval was obtained from the independent Medical Ethics Committee of the University Hospital Ghent and Ghent University. Participants provided written informed consent before participation, in which they were informed about the study’s purpose, procedures, potential risks, and their right to withdraw at any time without consequences. All collected data were pseudonymized to ensure confidentiality, and only pseudonymized data were used for analysis and publication.

## Funding sources

This work was supported by funding from the European Research Council (ERC) under the EU's Horizon 2020 Research and Innovation Program (grant agreement no 787,307).

## Data availability

Behavioural, fMRI, and RNN model data are available at OSF: https://osf.io/bz56g.

## Declaration of generative AI and AI-assisted technologies in the writing process

During the preparation of this work the first author used ChatGPT to refine sentence structure and enhance readability. After using this tool, the author reviewed and edited the content as needed and takes full responsibility for the content of the publication.

## CRediT authorship contribution statement

**Emmanouela Foinikianaki:** Writing – original draft, Visualization, Validation, Software, Methodology, Investigation, Formal analysis, Conceptualization. **Iris Ikink:** Writing – review & editing, Software, Investigation. **Thomas R. Colin:** Writing – review & editing, Software, Methodology, Formal analysis. **Ricardo J. Alejandro:** Writing – review & editing, Investigation. **Clay B. Holroyd:** Writing – review & editing, Supervision, Project administration, Funding acquisition, Conceptualization.

## Declaration of competing interest

The authors declare that they have no known competing financial interests or personal relationships that could have appeared to influence the work reported in this paper.

## Appendix Supplementary materials

Image, application 1

## Footnotes

1 We used cross-validated, squared Euclidean distance as our dissimilarity metric without multivariate noise normalization (MNN) as it has been argued that MNN does not consistently enhance reliability (Ritchie et al., 2021).
