# Supplementary material for: ACC representations of reward-driven motivation over hierarchically-organized behavior
Source: Neuroimage. 2025 Aug 15;317:121380. doi: 10.1016/j.neuroimage.2025.121380 (PMC12301845; doi:10.1016/j.neuroimage.2025.121380)
Supplement: Supplementary file 1 [file mmc1.docx]

***Supplementals of***

**ACC representations of reward-driven motivation over hierarchically-organized behavior**

Emmanouela Foinikianaki^1^*, Iris Ikink^1^, Thomas R. Colin^1^, Ricardo J. Alejandro^1^, Clay B. Holroyd^1^

^1^Department of Experimental Psychology, Ghent University, Ghent, Belgium

*Corresponding author: Emmanouela Foinikianaki, [emmanouela.foinikianaki@ugent.be](about:blank)

*1. Univariate Analysis & Results*

First, contrasts of interest were computed for each step of the Rewarded and the Non-rewarded sequences to generate two Reward contrasts (Reward > Non-reward), one for step 1 only (corresponding to the initial instruction phase of the sequence) and another one for all steps 1-6 (corresponding to the entire period of task execution). The voxel-level threshold was set to 0.001 uncorrected, using a whole-brain cluster-level familywise error (FWE) cluster correction of 0.05.

For the univariate analysis, we ran two different tests on the activation brain maps, one for step 1, which cued the participants for either a reward or a non-rewarded trial, and one for all of the steps 1 to 6. The Reward contrast (Reward > Non-reward, Suppl. Fig. 2A, Suppl. Table 2A) for step 1 revealed significant activation in right nucleus accumbens, right anterior cingulate cortex (ACC, supracallosal), left pulvinar medial, right middle frontal gyrus, right middle cingulate & paracingulate gyri, right & left cerebellar hemisphere and precentral & postcentral gyri. The Reward contrast (Reward > Non-reward, Suppl. Fig. 2B, Suppl. Table 2B) for all steps 1-6 revealed significant activation in right caudate nucleus, right insula, right middle cingulate gyrus, left middle frontal gyrus, left inferior parietal gyrus and lobule VIII of vermis.

*2. Exploratory Analysis on the reward effects in other brain regions*

For the purpose of comparison, we explored the reward-related effects observed in the next 3 most significant clusters identified by the RNN-RDM RSA, namely, the right inferior occipital gyrus (219 voxels, x=24, y=-94, z=-1) (Suppl. Fig. 1A), the left posterior crus I of cerebellum lobe (142 voxels, x=-39, y=-58, z=-34) (Suppl. Fig. 1D), and the left precuneus of the parietal lobe (139 voxels, x=-6, y=-73, z=41) (Suppl. Fig. 1G) (Suppl. Table 2C). For each of these clusters we repeated the ROI classification and statistical analysis that we applied to the ACC cluster, constructing a 24x24 RDM for the 24 different states using the cross-validated Euclidean distance as the distance metric (Suppl. Fig. 1B, 1E, 1H). A three-way repeated measures ANOVA was performed to evaluate the effects of condition (reward), step progression and task on the distances separately for each ROI (Suppl. Table 4).

Notably, unlike the ACC - which was completely insensitive to differences between the coffee and tea tasks (F=.003, p=0.9532) - both the occipital gyrus and the precuneus exhibited a strong main effect of task (coffee versus tea, F= 12.59, p<0.001 and F=8.14, p<0.01, respectively). Furthermore, posterior cerebellum showed a main effects of reward condition and step progression but no interactions, suggesting that the effect of reward on the representations extended beyond the beginning of the sequence to all of the steps in the sequence (Suppl. Table 4A-C).


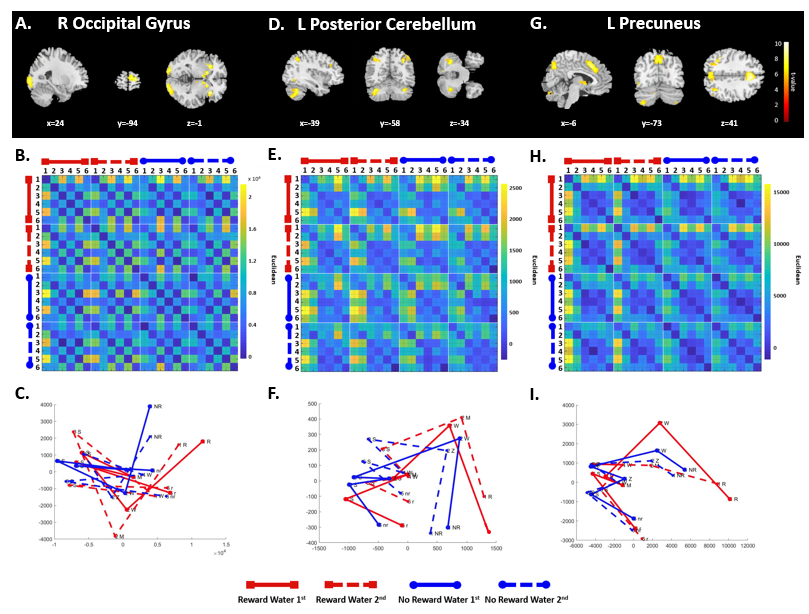


*Suppl. Figure 1*

**Exploratory Analysis of the next 3 most significant clusters identified by RNN-RDM RSA.** (A-C) Right occipital gyrus (coordinates, x = 28, y = -84, z = 1). (D-F) left posterior cerebellum (coordinates, x = 39, y = -58, z = -34). (G-I) left precuneus (coordinates, x = 6, y = -73, z = 41). For each region (top row), the RDMs (middle row) and MDS plots (bottom row) are derived from the Euclidean distances of all pairwise combinations of task steps for the rewarded and non-rewarded conditions (arbitrary units).


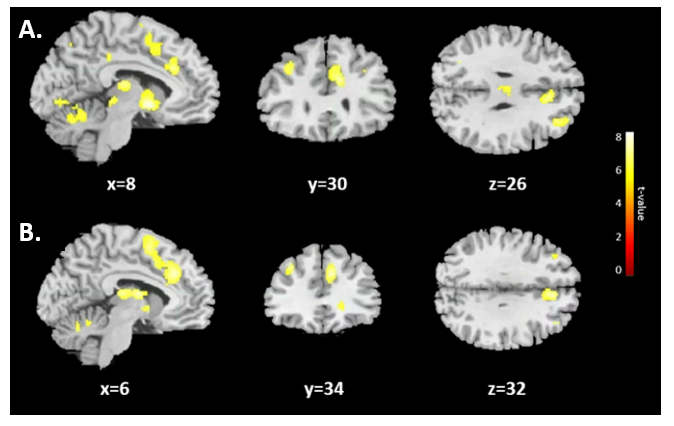


*Suppl. Figure 2*

**Univariate Analysis Results.** (A) Univariate contrast for Step 1, comparing Rewarded vs. Non-rewarded conditions. (B) Univariate contrast across all steps (1-6), comparing Rewarded vs. Non-rewarded conditions. Significant activations are shown in brain regions including the nucleus accumbens, anterior cingulate cortex (ACC), pulvinar, and middle cingulate cortex, highlighting the reward-related activity differences at the initial task cue (Step 1) and throughout the entire task sequence.

Suppl. Table 1

***Structured sequence of actions required for the "tea (water 1st)" task.*** This table presents the goals, inputs, and outputs at each step of the "tea (water 1st)" sequence, detailing the actions required to complete the task successfully.

| **Step** | **Goal** | **Input** | **Target output** |
| --- | --- | --- | --- |
| 1 | Tea (water 1st) | Make tea | Add teabag |
| 2 | Tea (water 1st) | Add teabag | Add water |
| 3 | Tea (water 1st) | Add water | Stir |
| 4 | Tea (water 1st) | Stir | Add sugar |
| 5 | Tea (water 1st) | Add sugar | Stir |
| 6 | Tea (water 1st) | Stir | Serve tea |

Suppl. Table 2

**Activation value maps for the Univariate Analysis, Representational Similarity Analysis (RSA) and Classification Analysis (CA)**. This table presents the activation values for key brain regions identified during univariate analysis and RSA classification, comparing Rewarded and Non-rewarded conditions across different steps in the task sequence.

|  | ***MNI Coordinates*** | | | | ***Cluster*** | ***Peak*** | ***cluster level*** |
| --- | --- | --- | --- | --- | --- | --- | --- |
| ***Region*** | **x** | **y** | **z** |  | **Size** | **T** | ***p-value*** |
| ***A. Univariate Analysis, Reward > NonReward Step 1*** |  |  |  |  |  |  | ***p(FWE-cor)*** |
| Nucleus Accumbens, right | 8 | 6 | -6 |  | 609 | 8.22 | 0.000 |
| Anterior cingulate cortex, supracallosal, right | 8 | 30 | 26 |  | 790 | 7.58 | 0.000 |
| Pulvinar medial, left | -12 | -28 | 10 |  | 323 | 7.38 | 0.000 |
| Middle frontal gyrus, right | 34 | 38 | 36 |  | 192 | 7.07 | 0.000 |
| Middle cingulate & paracingulate gyri, right | 2 | -30 | 38 |  | 177 | 7.05 | 0.000 |
| Crus I of cerebellar hemisphere, right | 40 | -58 | -32 |  | 1030 | 6.97 | 0.000 |
| Crus I of cerebellar hemisphere, left | -44 | -64 | -30 |  | 380 | 6.85 | 0.000 |
| Lobule VIII of cerebellar hemisphere, right | 20 | -66 | -46 |  | 227 | 6.52 | 0.000 |
| Postcentral gyrus, left | -46 | -28 | 52 |  | 152 | 6.48 | 0.000 |
| Precentral gyrus, left | -36 | -12 | 64 |  | 130 | 6.35 | 0.001 |
|  |  |  |  |  |  |  |  |
| ***B. Univariate Analysis, Reward > NonReward Steps 1-6*** | |  |  |  |  |  | ***p(FWE-cor)*** |
| Caudate nucleus, right | 12 | -2 | 14 |  | 1178 | 7.98 | 0.000 |
| Insula, right | 42 | 24 | -4 |  | 477 | 7.64 | 0.000 |
| Middle cingulate & paracingulate gyri, right | 6 | 34 | 32 |  | 699 | 7.43 | 0.000 |
| Middle frontal gyrus, left | -34 | 32 | 40 |  | 100 | 7.08 | 0.000 |
| Inferior parietal gyrus, excluding supramarginal and angular gyri, left | -56 | -34 | 46 |  | 385 | 6.81 | 0.000 |
| Lobule VIII of vermis | 2 | -64 | -22 |  | 364 | 6.66 | 0.000 |
|  |  |  |  |  |  |  |  |
| ***C. Searchlight Representational Similarity Analysis*** |  |  |  |  |  |  | ***p(FWE-cor)*** |
| Middle cingulate & paracingulate gyri, right | 3 | 29 | 35 |  | 310 | 10.47 | 0.000 |
| Middle occipital gyrus, left | -18 | -100 | 5 |  | 128 | 9.35 | 0.000 |
| Inferior occipital gyrus, right | 24 | -94 | -1 |  | 219 | 9.13 | 0.000 |
| Crus I of cerebellar hemisphere, left | -39 | -58 | -34 |  | 142 | 9.07 | 0.000 |
| Angular gyrus, right | 33 | -58 | 44 |  | 121 | 8.63 | 0.000 |
| Precuneus, left | -6 | -73 | 41 |  | 139 | 8.04 | 0.000 |

Continuation Suppl. Table 2

| ***D. Searchlight Classification***  ***Analysis (Reward vs nonReward)*** | | |  |  |  |  | ***p(uncorr.)*** |
| --- | --- | --- | --- | --- | --- | --- | --- |
| Lobule VIII of cerebellar hemisphere, right | 12 | -64 | -49 |  | 134 | 5.41 | 0.004 |
| Superior frontal gyrus, dorsolateral, right | 18 | 62 | 11 |  | 229 | 4.68 | 0.000 |
| Superior frontal gyrus, medial, right | 9 | 62 | 11 |  | 229 | 4.68 | 0.000 |
| Lobule VI of vermis | 3 | -67 | -10 |  | 278 | 4.65 | 0.000 |
| Insula, right | 33 | 23 | 5 |  | 274 | 4.61 | 0.000 |
| Middle cingulate & paracingulate gyri, right | -6 | 14 | 41 |  | 188 | 4.23 | 0.001 |
|  |  |  |  |  |  |  |  |

Suppl. Table 3

***Post-hoc step distances focusing on Step 1 relative to other steps.*** This table presents the pairwise comparisons of step distances in caudal ACC for the rewarded and non-rewarded conditions, highlighting significant differences between Step 1 and subsequent steps of the task sequence.

| Steps | Estimate | df | t.ratio | p.value |
| --- | --- | --- | --- | --- |
| Step 1 to 2 | 996.57 | 7007 | 2.18 | **0.029** |
| Step 1 to 3 | 1394.31 | 7007 | 3.05 | **0.002** |
| Step 1 to 4 | 797.04 | 7007 | 1.74 | 0.081 |
| Step 1 to 5 | 1706.68 | 7007 | 3.74 | **0.000** |
| Step 1 to 6 | 371.9 | 7007 | 0.81 | 0.416 |

Suppl. Table 4

***Three-way repeated measures ANOVA results on step distances in the next three brain regions identified by model-based RSA.*** A three-way repeated-measures ANOVA was conducted on the distances for each step of the four task sequences, with Reward condition (Rewarded vs. Non-rewarded), Step (1 to 6), and Task (coffee vs. tea) as factors. The analysis was performed for four brain regions: the occipital gyrus, posterior cerebellum, and precuneus.

|  |  |  |  |  |  |
| --- | --- | --- | --- | --- | --- |
| **A. Inferior Occipital Gyrus** | | | | | |
|  | df | Sum of Squares | Mean of Squares | F-value | p_value_ |
| **Condition** | **1** | **2.21E+09** | **2.21E+09** | **24.988** | **6.06E-07 ***** |
| **Step** | **35** | **8.71E+10** | **2.49E+09** | **28.163** | **<2E-16 ***** |
| **Task** | **1** | **1.11E+09** | **1.11E+09** | **12.586** | **0.000394 ***** |
| **Condition * Step** | **35** | **5.48E+09** | **1.57E+08** | **1.772** | **0.003464 **** |
| Step * Task | 35 | 4.40E+09 | 1.26E+08 | 1.425 | 0.050612 . |
| Condition * Step * Task | 35 | 3.06E+09 | 8.74E+07 | 0.989 | 0.4863 |
| Residuals | 3408 | 3.01E+11 | 8.83E+07 |  |  |
|  |  |  |  |  |  |
| **B. Posterior Cerebellum** | | | | | |
|  | df | Sum of Squares | Mean of Squares | F-value | p_value_ |
| **Condition** | **1** | **1.36E+07** | **1.36E+07** | **4.901** | **0.0269 *** |
| **Step** | **35** | **1.34E+09** | **3.83E+07** | **13.818** | **<2E-16 ***** |
| Task | 1 | 6.51E+05 | 6.51E+05 | 0.235 | 0.6278 |
| Condition * Step | 35 | 1.03E+08 | 2.93E+06 | 1.058 | 0.9359 |
| Step * Task | 35 | 6.42E+07 | 1.84E+06 | 0.663 | 0.9359 |
| Condition * Step * Task | 35 | 3.03E+07 | 8.66E+05 | 0.313 | 1 |
| Residuals | 3408 | 9.43E+09 | 2.77E+06 |  |  |
|  |  |  |  |  |  |
| **C. Precuneus** | | | | | |
|  | df | Sum of Squares | Mean of Squares | F-value | p_value_ |
| **Condition** | **1** | **1.74E+09** | **1.74E+09** | **27.05** | **2.1E-07 ***** |
| **Step** | **35** | **4.59E+10** | **1.31E+09** | **20.376** | **<2E-16 ***** |
| **Task** | **1** | **5.24E+08** | **5.24E+08** | **8.144** | **0.00435 **** |
| **Condition * Step** | **35** | **3.60E+09** | **1.03E+08** | **1.598** | **0.01433 *** |
| Step * Task | 35 | 1.39E+09 | 3.97E+07 | 0.618 | 0.96231 |
| Condition * Step * Task | 35 | 1.88E+09 | 5.36E+07 | 0.832 | 0.74595 |
| Residuals | 3408 | 2.19E+11 | 6.44E+07 |  |  |
